# Risk Factors for Herpes Zoster in Patients with Chronic Kidney Disease: A Case-Control Study

**DOI:** 10.3390/vaccines9090963

**Published:** 2021-08-28

**Authors:** Zhenxing Li, Qiao Wang, Jiahui Ma, Zhi Li, Dong Huang, Yuzhao Huang, Haocheng Zhou

**Affiliations:** 1 Department of Pain, Institute of Pain Medicine, Third Xiangya Hospital of Central South University, Changsha 410013, China; Zhenxing_Li@csu.edu.cn (Z.L.); 198312208@csu.edu.cn (Q.W.); 198302074@csu.edu.cn (J.M.); huangdong6619@vip.163.com (D.H.); 2 Department of Nephrology, Third Xiangya Hospital of Central South University, Changsha 410013, China; lizhi080108@sina.com; 3 Hunan Key Laboratory of Brain Homeostasis, Central South University, Changsha 410013, China; 4 Department of Orthopedics, Third Xiangya Hospital of Central South University, Changsha 410013, China; hyzxiangya3@outlook.com

**Keywords:** chronic kidney disease, herpes zoster, risk factor, case-control

## Abstract

(1) Background: Chronic kidney disease (CKD) increases the susceptibility to the presence of herpes zoster (HZ). Little is known about the risk factors of HZ in CKD patients; (2) Methods: This is a case-control study. CKD patients diagnosed with HZ between January 2015 and June 2021 in a tertiary hospital were identified. One age- and gender- matched control was paired for each case, matched to the date of initial HZ diagnosis. Conditional multiple logistic regression was used to evaluate the risk factors associated with the presence of HZ; (3) Results: Forty-seven HZ patients and controls were identified. In general, about 73.40% (69 out of 94) patients were classified at IV to V stages of CKD. Immunosuppressive agents (*p* = 0.0012) and dialysis therapy (*p* = 0.021) were reported more frequently in the HZ cohort. Compared with the control group, the total white cell count and lymphocyte count were significantly lower in the HZ group (*p* value of 0.032 and 0.003, respectively). The conditional logistics regression model revealed that previous immunosuppressants administration (odds ratio: 10.861, 95% CI: 2.092~56.392, *p* = 0.005) and dialysis therapy (odds ratio: 3.293, 95% CI: 1.047~10.355, *p* = 0.041) were independent risk factors of HZ in the CKD population; (4) Conclusions: Dialysis and immunosuppressants therapy were associated with greater risk of HZ disease in CKD patients. Further guideline may highlight the necessity of zoster vaccine for patients with CKD, who undertake associated treatment.

## 1. Introduction

The burden of chronic kidney disease (CKD) has become a great challenge for the global healthcare system, affecting almost 15% of adults in the United States [1]. As CKD progresses, costly therapy such as dialysis or kidney transplant may be required to maintain the function of kidney. Moreover, patients with CKD exhibit greater risk to developing cardiovascular disease, in both the dialysis- and non–dialysis dependent population [2]. Infectious disease is the second most common cause of morbidity and mortality in CKD, accounting for 30–36% of deaths [3,4]. The mechanism underlying the immune dysfunction of CKD includes poor nutritional condition, immunosuppressive medication, and uremic toxins [5]. Consequently, infectious-related mortality increased significantly in patients with CKD, especially for those who have progressed to end-stage renal disease (ESRD) [6].

Herpes zoster (HZ), also known as shingles, is a common viral disease that occurs with reactivation of the varicella-zoster virus. Accumulating evidence has suggested that CKD is an important risk factor for HZ [7,8,9,10,11,12]. Incidence of postherpetic neuralgia (PHN), the most common complication of HZ, also increases significantly among CKD cohort [13]. PHN patients often present physical, occupational, social, and psychosocial disabilities as a result of the unremitting pain [14]. Furthermore, the overall risk of adverse cardiovascular events also increased after the zoster attack [15,16]. Consequently, CKD patients who suffered HZ and HZ-related complications are prone to have extremely great risk of developing ESRD [17].

One effective approach to reduce the morbidity of HZ and PHN is to apply zoster vaccine [18]. Recent study has demonstrated that the zoster vaccine was effective against incident zoster for the elderly with CKD [19]. In addition to advancing ages, less is known about the other risk factors for HZ lesion in the CKD population. To achieve better clinical outcome of CKD, early recognition of potential HZ eruption and subsequent protective vaccine therapy is urgently needed. In the current study, we aim to examine the potential risk factors of zoster attack in CKD patients.

## 2. Materials and Methods

### 2.1. Study Population

The study was approved by the ethics committee of the Third Xiangya Hospital, Central South University (NO. 2050-s388), and informed consent was waived due to the observational design in this study.

Case: Ninety-two CKD patients diagnosed with HZ (ICD-10-CM codes: B02) between January 2015 and June 2021 at the Third Xiangya Hospital of Central South University were identified. Forty-five cases who underwent transplant were not included in this study.

Control: The controls were randomly retried from the remaining CKD patients, age- and sex- matched with the HZ cases. One control was identified for each case and matched to the date of initial herpetic diagnosis.

### 2.2. Data Collection

Two colleagues (Q.W. and Z.L.) independently reviewed the medical record of all cases and controls. One standard data collection form was applied to record the general characteristics and clinical information. The age of the patient was identified as the onset of the herpes rash. The first data available following admission were recorded and applied for further analysis. Previous immunosuppressants included long-term steroid (≥3 months, ≥5 mg per day) [20] and non-steroid medication. For non-steroid immunosuppressants, mycophenolate, cyclophosphamide, hydroxychloroquine, or triptolide may be used to treat CKD caused by systemic lupus erythematosus and nephrotic syndrome.

### 2.3. Statistical Analysis

Chi-squared test or Fisher exact test were conducted to compare categorical data. The Student’s *t*-test or Mann-Whitney U test was used when appropriate to analyze continuous data. Conditional multiple logistics analysis was performed to evaluate the independent risk factors associated with HZ disease in CKD patients. Variables with *p* values < 0.05 between cases and controls were included for multivariate logistics analysis. All continuous data are presented as mean ± standard deviation. Estimation of risk was presented as odds ratios (ORs) with 95% CIs, and two-tailed *p* value < 0.05 was considered statistically significant. All data analysis was processed with SPSS (version 26.0, Chicago, IL, USA).

## 3. Results

### 3.1. General Characteristics

The research of a medical database initially identified a total of 47 CKD cases with a diagnosis of HZ. Next, 47 age- and sex-matched controls were selected randomly from the remaining CKD cohort. Cardiovascular diseases were the most commonly reported comorbidities. The incidence of comorbidity was not significantly different between the HZ and non-HZ group. Likewise, the etiologies of CKD were similar in this study. Only 4 out of the 47 patients (8.51%) took regular immunosuppressive agents in the control group, and 38.30% (*n* = 18/47) for the HZ group, respectively (*p* = 0.0012). Compared with the control group, more patients required the renal replacement therapy of dialysis (70.21% versus 46.81%, *p* = 0.021). The majority of patients (73.40%) in this study were identified at stages IV to V of CKD. However, no significant difference of disease severity was found between groups (*p* = 0.859). In HZ group, the truncal sites were the mostly affected, accounting for approximately 62% of the cases. About 34% (16 out of 47) of HZ patients presented co-infection caused by bacterial or fungal pathogen. Ocular complications, including keratitis, iritis, retinitis, or visual impairment were not observed in those with ophthalmic involvement (*n* = 6). The general information of enrolled participants is given in Table 1.

### 3.2. Laboratory Result

The diagnostic details of the laboratory test are shown in Table 2. In general, the HZ patients presented a significant dysfunction of the immune system, characterized by reduced total lymphocyte account (*p* = 0.003) and white blood cell count (*p* = 0.032). No obvious difference of renal function was found between groups according to current data.

### 3.3. Logistic Regression

Compared with the control group, the patients who needed dialysis treatment had significant and higher risk of HZ (OR: 2.375; 95% CI: 1.040~5.425, *p* = 0.040). This significance remained (OR: 3.293; 95% CI: 1.047~10.355, *p* = 0.041) in the adjusted model (Table 3). Usage of immunosuppressant agent was also associated with increased risk for HZ in the conditional logistic model (OR: 10.861; 95%CI: 2.092~56.392, *p* = 0.005).

## 4. Discussion

In this case-control study, we investigated 94 patients with CKD at a tertiary hospital and aimed to evaluate the potential risk factors for HZ eruption. To our knowledge, it is the first time we identified the CKD-related therapy as independent risk factors of HZ attack in case-control design.

Kidney disease severity is classified into five stages according to the level of the glomerular filtration rate [21]. A previous study has demonstrated that ESRD is a risk factor of HZ [10]. Similarly, we found that most of the HZ cases (72.34%, n = 34/47) were identified at the stages IV to V of CKD in this study. The overall incidence of ESRD increases with age, and the majority of patients who reach ESRD are 65 years or older [22]. Despite disease severity, herpes zoster is also of particular concern in the elderly [23,24]. The mean age of subjects in this study was around 60 years old. Given the age-matched case-control design of this study, we cannot evaluate the impact of advancing age on the risk for HZ. It is necessary to examine the relationship between age and HZ susceptibility in CKD cohort with a larger sample size in the future.

Consistent with previous reports, our data indicated that patients who regularly take immunosuppressive medication were more likely to have zoster disease [25,26]. The common co-morbidity of CKD patients who use immunosuppressive therapy, includes nephrotic syndrome, rheumatoid arthritis, and systemic lupus erythematosus. As the representative immunosuppressant agent, long-term usage of steroid (≥3 months, ≥5 mg per day) [20] was frequently found in the HZ cohort. Our finding is consistent with steroid-associated side effects of infectious complications [27].

Despite medication treatment, we found that dialysis was an independent risk factor of zoster lesion. There were 33 out of 47 cases (70.21%) treated with dialysis, and 46.81% of the control group, respectively. The large cohort study conducted by Lin et al. showed similar results, that both peritoneal dialysis and hemodialysis patients presented a higher incidence of HZ compared with those without chronic renal disease [28]. The higher incidence of HZ was reported in patients who underwent renal transplant [28,29]. Although we identified 46 HZ cases after renal transplant in the initial research. We did not enroll these patients due to the complex factors in the status of renal transplant.

Several factors may contribute to the susceptibility of HZ attack in CKD cohort treated by dialysis. First, loss of proteins with immune role (immunoglobulin, zinc-binding protein, and ferritin) is associated with the generalized immunosuppressive status in CKD [30]. In addition, the disruption of protective cutaneous barrier caused by peritoneal or vascular access is a potential risk factor for infectious disease [31]. The initial onset of dialysis therapy during advanced CKD is also associated with high risk of infection and other adverse events [32]. Moreover, the exposure to viral/bacterial pathogen increases with the number of medical visits for dialysis treatment [33].

There are some correlations between the immune deficiency and the incidence of infectious complications in CKD patients, characterized by a significant lymphopenia [34]. Similarly, we found that the total lymphocyte account was significantly lower in HZ patients compared with the control group. The mechanism underlying the lymphopenia in CKD is the lower T cell homeostatic proliferation [35]. It is not surprising that total leukocyte counts were kept in the normal range between groups, mainly due to routine medication to prevent leukopenia. Thus, a combination of mild neutrophilia and significant lymphopenia potentially caused an increased neutrophil-to-lymphocyte ratio [34].

Corroborative evidence in CKD population has suggested an inverse relationship between serum albumin and poor prognosis [36,37]. However, the context in herpetic lesion remains unclear. In our study, we did not find a significant reduction of serum albumin in CKD patient with HZ, compared with the control group. The allocation of CKD patients based on serum albumin levels is helpful in prediction of infection-related death, but not available in this study due to limited number of subjects [37].

Our study has some limitations beyond the limited sample size. First, the retrospective nature of this study design is likely to omit the feature data. However, our data may be helpful in long-term management of CKD patients due to the case-control study design. The study design can help avoid potential bias such as CKD severity, age, gender, and other related factors. In addition, the data we collected were derived from general characteristics and routine laboratory test. Specific examination of immune function such as lymphocyte subset analysis and interleukin-2 may be useful for the assessment of immune function in CKD patients [34,38]. It is necessary to conduct a further prospective study with a larger cohort size to examine the other potential risk factors of HZ in the CKD population. Finally, information regarding the patient’s course after discharge was not available for the control group. This supports the need for future research to conduct long-term follow-up.

In conclusion, dialysis and immunosuppressants therapy independent risk factors were associated with the presence of HZ lesion in patients with CKD. Further guidelines may highlight the necessity of the zoster vaccine for patients with CKD, who regularly undertake these CKD-related treatment.

## Figures and Tables

**Table 1:** General characteristics of herpetic and non-herpetic patients with CKD.

Variables	HZ ^1^	Non-HZ	*p* Value
N	47	47	
Age (years)	59.11 ± 13.78	56.79 ± 13.75	0.416
Sex (female, %)	21 (45.6)	21 (45.6)	>0.999
Body Mass Index (kg/m^2^)	21.96 ± 3.16	22.44 ± 3.28	0.478
Comorbidities (n, %)			
Cardiovascular disease	43, (91.49)	37, (78.72)	0.146
Diabetes mellitus	9, (19.15)	7, (14.89)	0.583
Respiratory disease	16, (34.04)	16, (34.04)	>0.999
Neoplasm	1, (2.13)	0	>0.999
CKD etiology (n, %)			
Diabetic nephropathy	6, (12.77)	5, (10.64)	0.748
Ischemic/hypertensive nephropathy	11, (23.40)	8, (17.02)	0.441
Chronic glomerulonephritis	22, (46.81)	25, (53.19)	0.536
Other/unknown	9, (19.15)	9, (19.15)	>0.999
Previous intervention (n, %)			
Immunosuppressants	18, (38.30)	4, (8.51)	0.0012 **
Dialysis therapy	33, (70.21)	22, (46.81)	0.021 *
CKD stage (n, %)			0.859
I	0	0	
II	6, (12.77)	5, (10.64)	
III	7, (14.89)	7, (14.89)	
Ⅳ	6, (12.77)	9, (19.15)	
Ⅴ	28, (59.57)	26, (55.32)	
Anatomical site of zoster (n, %)			
Trigeminal	6, (12.77)		
Truncal	29, (61.70)		
Upper/lower extremity	12, (25.53)		
Site unspecified	4, (8.51)		
Short-term complication of HZ (n, %)			
Co-infection	16, (34.04)		
Ophthalmic complication	0		
Disseminated HZ	2, (4.26)		
Neurologic complication	0		

^1^ HZ: Herpes Zoster; CKD: Chronic Kidney Disease; *: *p* < 0.05; **: *p* < 0.01.

**Table 2:** Laboratory findings of CKD patients.

Laboratory Indicators	HZ	Non-HZ	*p* Value
White blood cell count, 10^9^/L	6.38 ± 2.40	7.54 ± 2.75	0.032 *
Neutrophil count, 10^9^/L	4.84 ± 2.18	5.64 ± 2.63	0.114
Lymphocyte count, 10^9^/L	0.93 ± 0.49	1.25 ± 0.51	0.003 **
Eosnophils count, 10^9^/L	0.14 ± 0.14	0.20 ± 0.49	0.422
Basophil count, 10^9^/L	0.02 ± 0.02	0.03 ± 0.03	0.072
Monocyte count, 10^9^/L	0.44 ± 0.26	0.42 ± 0.18	0.701
Platelet count, 10^9^/L	173.55 ± 85.24	179.76 ± 69.56	0.701
Red blood cell count, 10^12^/L	3.08 ± 0.76	3.08 ± 0.85	0.963
Hb (g/L)	93.70 ± 22.75	92.81 ± 25.51	0.858
Hematocrit (%)	28.88 ± 6.71	28.68 ± 7.49	0.891
Neutrophils-lymphocytes ratio (%)	6.93 ± 5.41	5.51 ± 4.35	0.164
C-reactive protein (mg/L)	21.59 ± 45.38	14.72 ± 35.44	0.576
Erythrocyte sedimentation rate (mm/h)	40.43 ± 26.93	48.77 ± 33.30	0.233
Glucose (mmol/L)	5.37 ± 2.05	5.29 ± 1.69	0.831
Procalcitonin (ng/mL)	0.99 ± 0.97	0.72 ± 1.66	0.598
Serum albumin (g/L)	31.82 ± 6.10	33.84 ± 6.64	0.126
Serum globulin (g/L)	25.02 ± 6.08	24.91 ± 4.77	0.925
Ratio of albumin to globulin	1.34 ± 0.39	1.39 ± 0.36	0.486
Alanine aminotransferase (U/L)	18.51 ± 13.36	18.84 ± 13.89	0.907
Aspartate aminotransferase (U/L)	20.26 ± 15.35	21.67 ± 10.08	0.616
Total bilirubin (umol/L)	7.60 ± 5.21	8.52 ± 5.52	0.415
Direct bilirubin (umol/L)	2.38 ± 3.28	2.17 ± 2.16	0.728
Total bile acid (umol/L)	4.97 ± 5.64	3.68 ± 2.88	0.177
Low-density lipoprotein (mmol/L)	2.13 ± 0.76	2.27 ± 1.11	0.490
High-density lipoprotein (mmol/L)	1.17 ± 0.40	1.28 ± 0.50	0.227
Total cholesterol (mmol/L)	4.49 ± 1.23	4.56 ± 1.77	0.831
Triglyceride (mmol/L)	2.19 ± 1.84	1.81 ± 2.05	0.356
Urea (mmol/L)	32.74 ± 76.42	20.60 ± 10.24	0.286

*: *p* < 0.05; **: *p* < 0.01.

**Table 3:** Association between incidence of herpes zoster and the selected variables.

Covariates	Not Adjusted OR (95% CI)	*p* Value	Adjusted OR (95% CI)	*p* Value
Immunosuppresants	5.667 (1.661~19.336)	0.006 **	10.861 (2.092~56.392)	0.005 **
Dialysis therapy	2.375 (1.040~5.425)	0.040 *	3.293 (1.047~10.355)	0.041 *
White blood cell count	0.825 (0.690~0.986)	0.034 *	0.830 (0.645~1.068)	0.148
Lymphocyte count	0.299 (0.118~0.758)	0.011 *	0.579 (0.178~1.878)	0.362

*: *p* < 0.05; **: *p* < 0.01.

## Data Availability

The data presented in this study are available on request from the corresponding author. The data are not publicly available due to privacy and ethical reasons.
